# Supplementary material for: Multiomic profiling of ER-positive HER2-negative breast cancer reveals markers associated with metastatic spread
Source: Breast Cancer Res. 2026 Jan 12;28:12. doi: 10.1186/s13058-025-02173-9 (PMC12810005; doi:10.1186/s13058-025-02173-9)
Supplement: Supplementary file 5 — Supplementary Information: Supplementary Figures S1-S7 [file 13058_2025_2173_MOESM5_ESM.pdf]

Supplementary Information

Multiomic profiling of ER-positive HER2-negative breast cancer reveals markers associated with metastatic spread

Sergio Mosquim Junior, Måns Zamore, Johan Vallon-Christersson, Lisa Rydén, Fredrik Levander

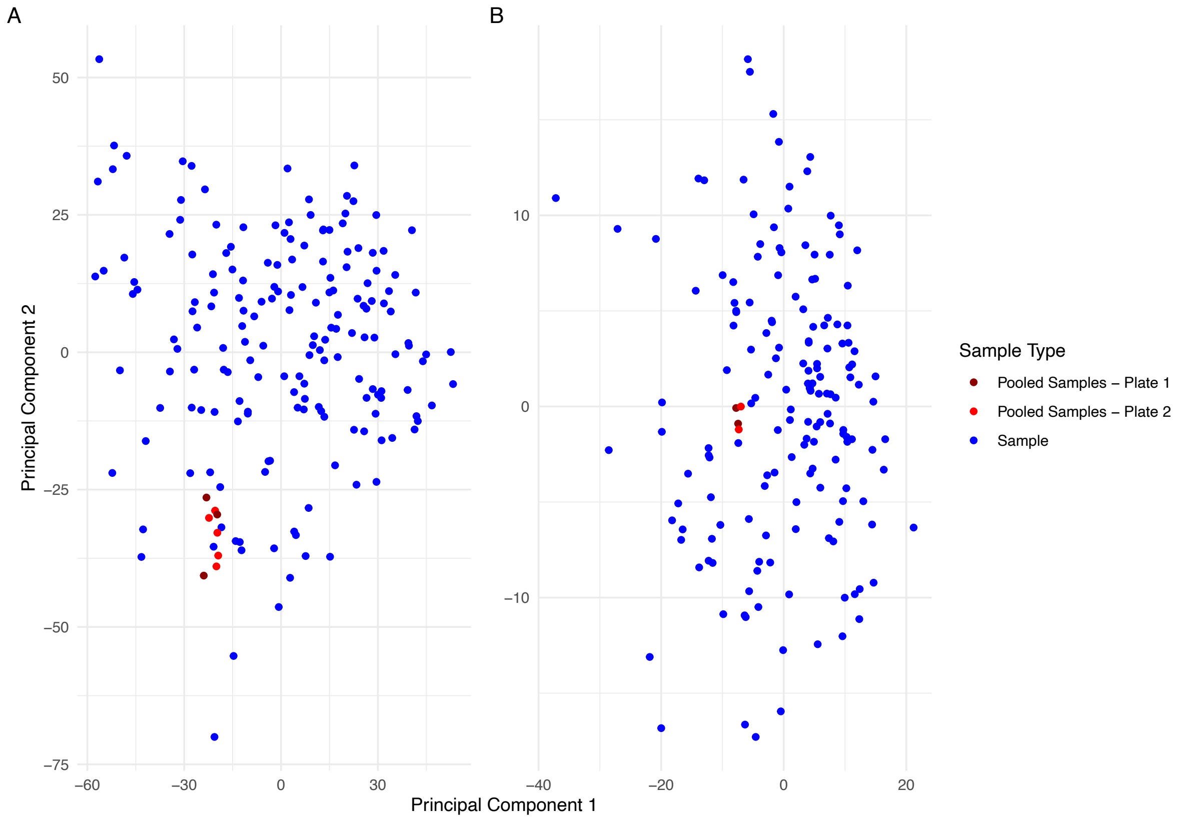

Supplementary Figure S1 Plot of the two first principal components of resulting from Principal Component Analysis (PCA) using **(A)** full proteome data and **(B)** phosphoproteome data.

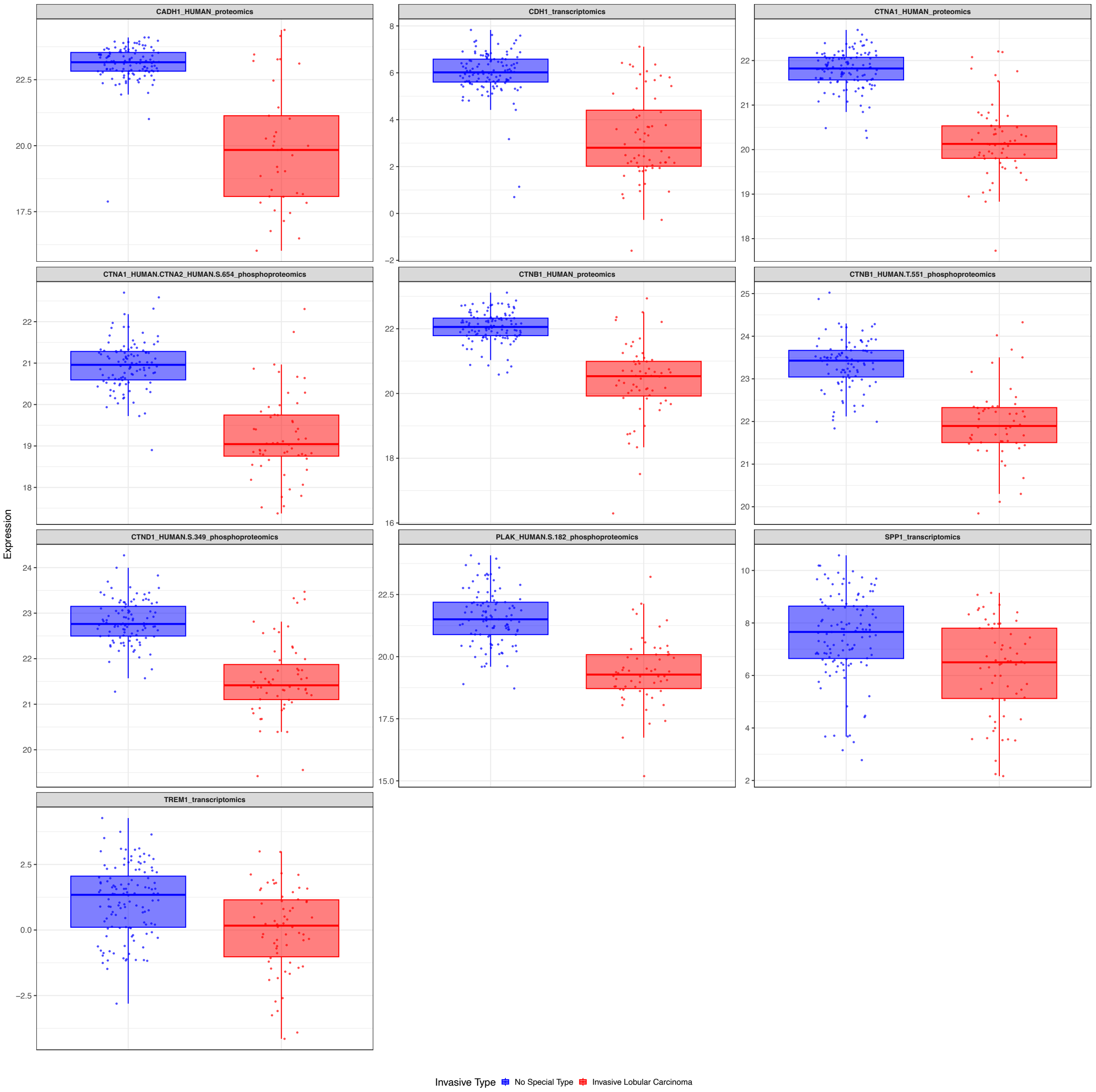

Supplementary Figure S2 Boxplot of the top most significant unique differentially expressed features in the three omics comparing NST and ILC.

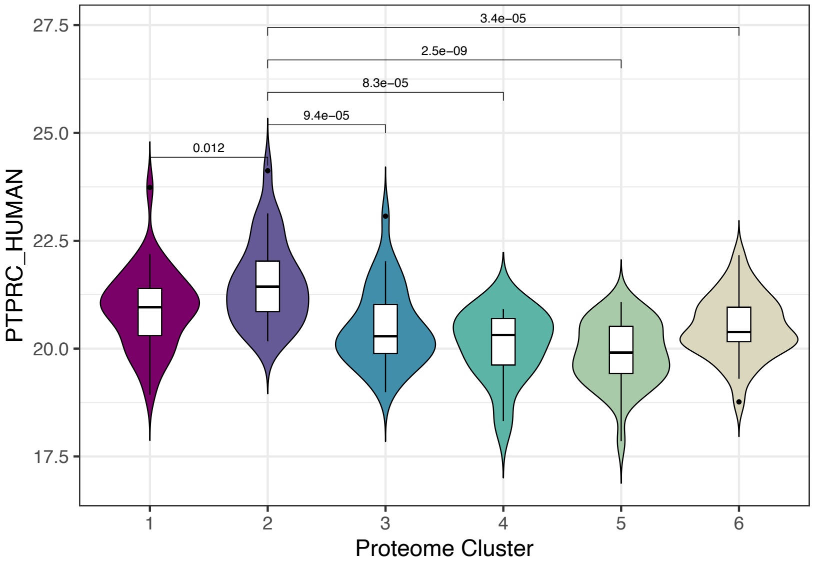

Supplementary Figure S3 Violin plots comparing CD45 (PTPRC_HUMAN protein) across the different proteome clusters. Individual p-values from Student's t-test for comparisons with cluster 2 are written out in the figure.

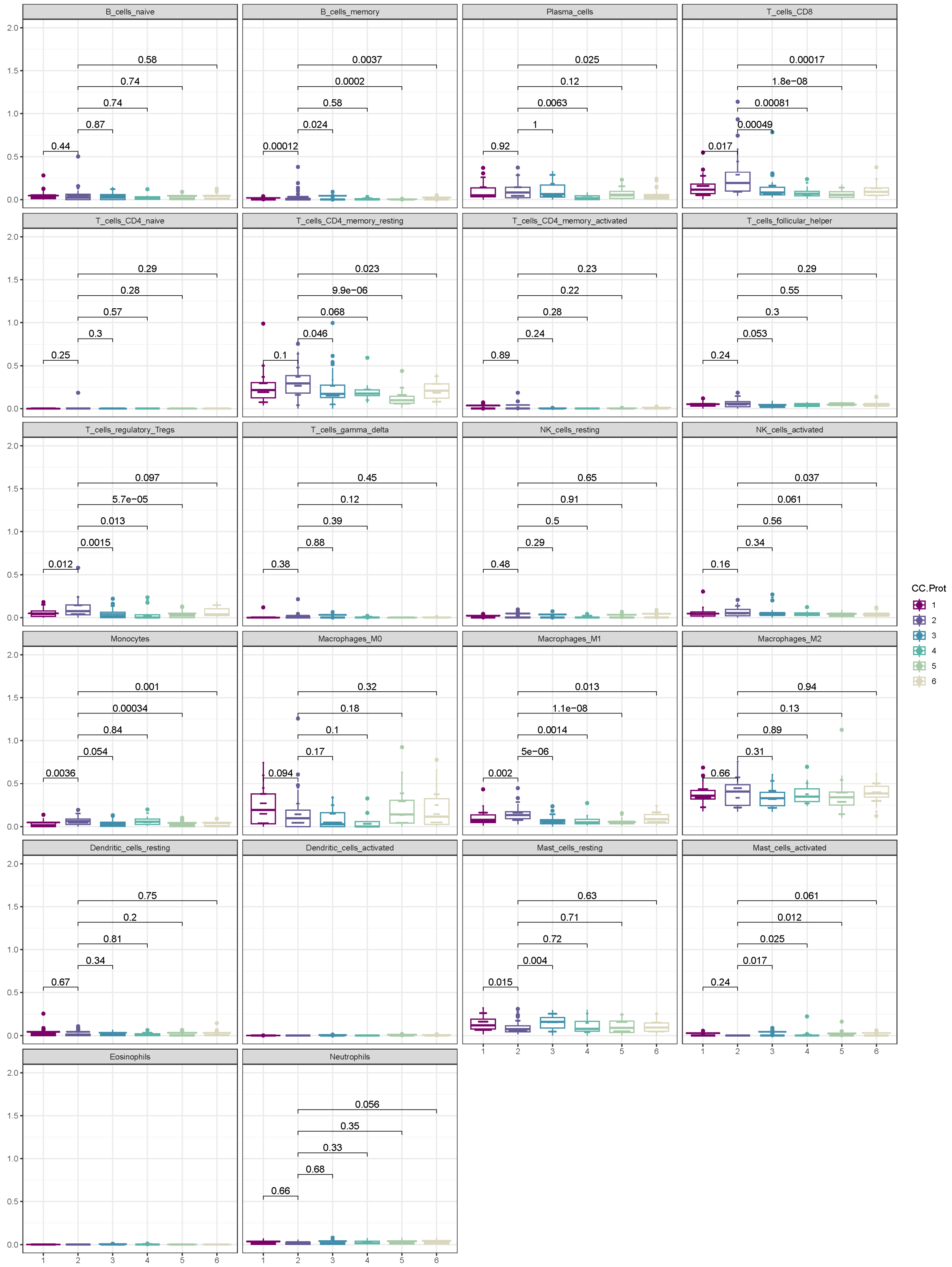

Supplementary Figure S4 Comparison of immune infiltration estimates based on CIBERSORTx using absolute mode and LM22 signature matrix. P-values for Wilcoxon test comparisons with cluster 2 are written out in the figures.

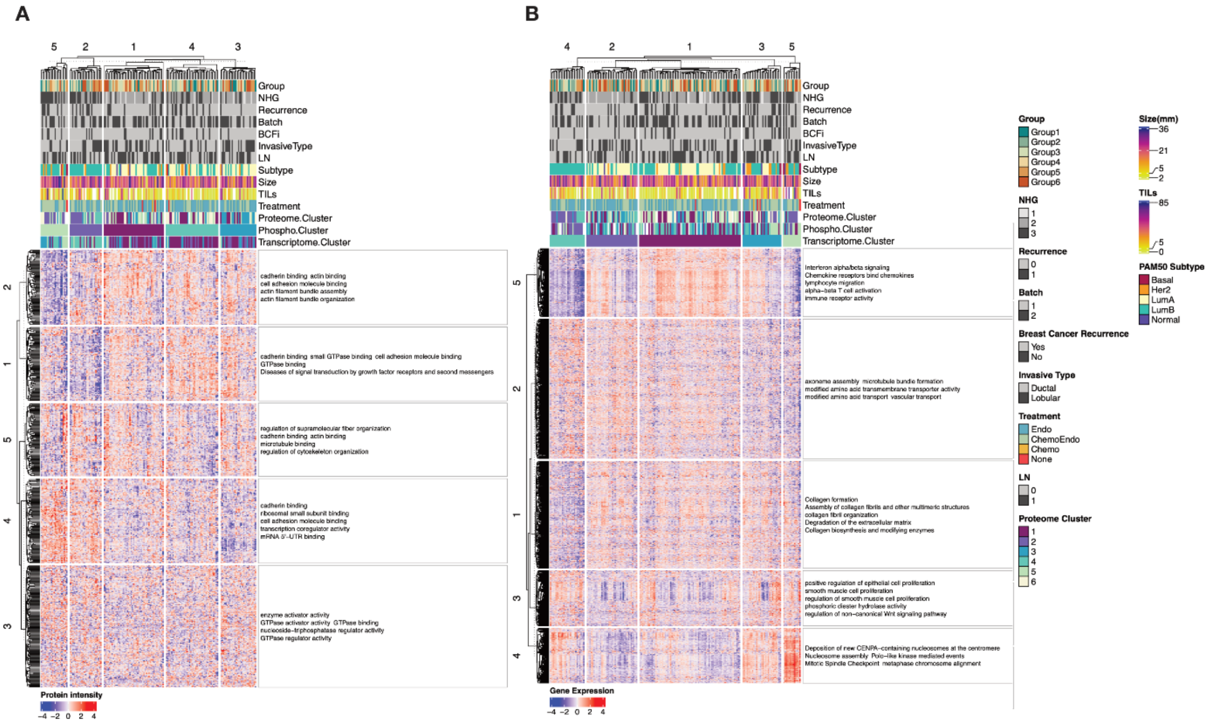

Supplementary Figure S5 Consensus clustering at the phosphoproteome **(A)** and transcriptome **(B)** level. The features were further clustered in 5 distinct clusters based on K-means and the terms represent enrichment based on G:profiler.

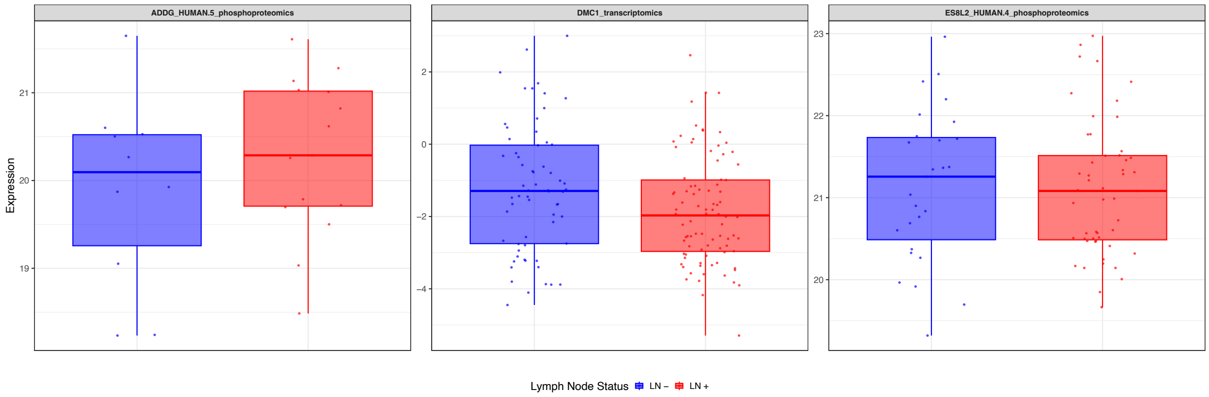

Supplementary Figure S6 Boxplot of features associated with lymph node metastasis passing multivariable Cox proportional hazards model (multivariable p-value <0.05) with RFS as outcome.

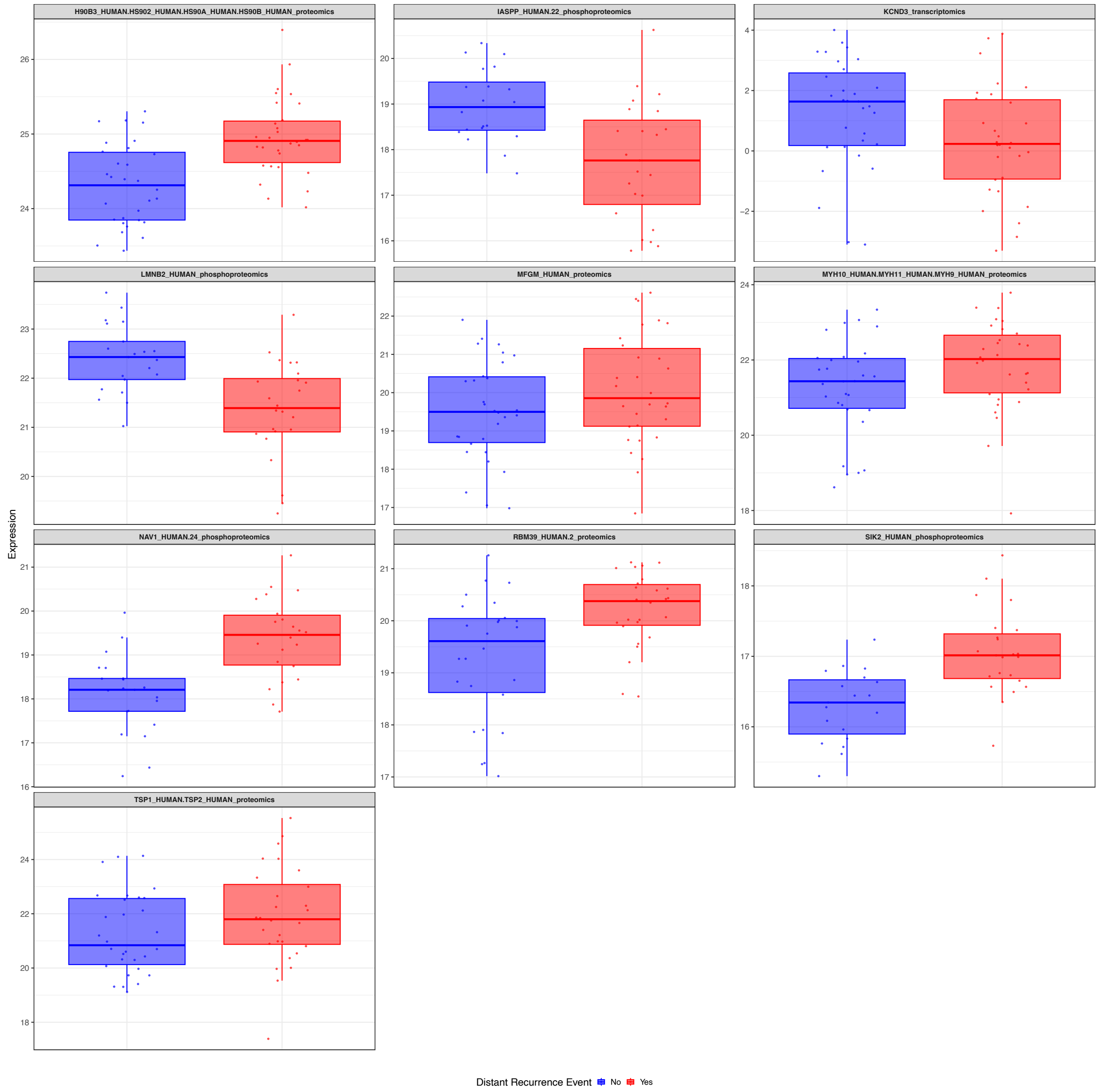

Supplementary Figure S7 Boxplot of features associated with distant metastasis passing multivariable Cox proportional hazards model (multivariable p-value <0.05) with DRFS as outcome.
